# Everolimus-eluting bioresorbable scaffolds for treatment of coronary artery disease in patients with diabetes mellitus: the midterm follow-up of the prospective ABSORB DM Benelux study

**DOI:** 10.1186/s12933-019-0827-z

**Published:** 2019-03-09

**Authors:** T. M. Hommels, R. S. Hermanides, S. Rasoul, B. Berta, A. J. J. IJsselmuiden, G. A. J. Jessurun, E. Benit, B. Pereira, G. De Luca, E. Kedhi

**Affiliations:** 1 Isala Klinieken, Isala Hartcentrum, Dokter van Heesweg 2, 8025 AB Zwolle, The Netherlands; 2 Zuyderland Medisch Centrum, Heerlen, The Netherlands; 3 Albert Schweitzer Ziekenhuis, Dordrecht, The Netherlands; 4 Scheper Ziekenhuis, Emmen, The Netherlands; 5 Virga Jesse Ziekenhuis, Hasselt, Belgium; 6 Institut National de Chirurgie Cardiaque et de Cardiologie Interventionnelle, Luxembourg, Luxembourg; 7 Dokter van Heesweg 2, Postbus 10400, 8000 GK Zwolle, The Netherlands

**Keywords:** Bioresorbable scaffold, Diabetes mellitus, Coronary artery disease, Percutaneous coronary intervention, Scaffold thrombosis

## Abstract

**Background:**

Percutaneous coronary intervention (PCI) in patients with diabetes mellitus (DM) remains challenging even with modern drug-eluting stents (DES) due to high rates of repeat revascularization. Everolimus-eluting bioresorbable scaffolds (EE-BRS) might allow for repeat intervention prolonging the time interval of percutaneous treatment options.

**Methods:**

The ABSORB DM Benelux Study is a dedicated prospective, international study to evaluate the midterm safety and efficacy of EE-BRS in DM patients. All DM patients that received ≥ 1 EE-BRS for any indication were enrolled and prospectively followed. Study endpoints were major adverse cardiac events (MACE): a composite of all-cause death, any myocardial infarction (MI) and ischemic-driven target vessel revascularization (TVR); target lesion failure (TLF): a composite of cardiac death (CD), target vessel MI, and ischemic-driven target lesion revascularization (TLR), as well as definite or probable scaffold thrombosis (ScT).

**Results:**

Between April 2015 till March 2017, 150 DM patients and 188 lesions were treated and followed up to 3 years. Device implantation success was 100%. MACE occurred in 15.2% (event rate of 8.8 per 100 PY). TLF was reported in 11.7% (7.0 events per 100 PY). CD, target vessel MI, ischemic-driven TLR occurred in 3.4%, 3.6% and 5.5% respectively, while ScT was observed in 1.4%. There were no occurrences of late or very late ScT.

**Conclusion:**

EE-BRS treatment in DM patients shows comparable midterm safety and efficacy outcomes when historically compared with modern DES. New-generation EE-BRS might offer an attractive alternative to metallic DES in treatment of fast progressing atherosclerosis population as in DM patients.

*Trial registration* NTR5447. Registered 05 October 2015, retrospectively registered

## Introduction

Diabetes mellitus (DM) is the most common metabolic disorder worldwide and its incidence and prevalence is increasing [1, 2]. DM is considered an independent risk factor for coronary artery disease (CAD) and prognostic it is viewed as CAD equivalent [3–9]. CAD is responsible of 80% of the deaths and 75% of the hospital admissions in DM patients [10]. Despite the advantages of novel drug-eluting stent (DES) and improved medical regiments in DM treatment, high rate of restenosis remains a challenge [11]. Large randomized trials persistently show a trend towards higher rates of major adverse cardiovascular events in DM patients treated with percutaneous coronary intervention (PCI) compared to coronary artery bypass grafting (CABG) [12–14]. However, PCI is still largely performed in this high-risk population especially in young patients where long-term CABG outcomes are unknown.

The bioresorbable polymer drug-eluting scaffold ABSORB BVS is a coronary implantable device offering the potential of a temporary vessel scaffold combined with drug delivery capability. Considering the promising results from the everolimus-eluting bioresorbable scaffolds (EE-BRS) clinical trial programs, it was conceivable that EE-BRS implantation could be associated with favourable long-term outcomes compared to metallic DES, mainly because of its resorbable nature which in turn reduces the exposure to the inflammation trigger caused from the presence of foreign material in the vessel wall [15–22]. Particularly in DM patients, where diabetes-related chronic intravascular inflammation triggers a more aggressive restenosis, reduction of permanent inflammatory triggers may further improve clinical outcomes. Importantly, this device might permit a higher number of repetitive interventions as its not limited by lumen reducing multiple levels of metal, thus prolonging the time span where CAD could still be managed with PCI in these patients. The evidence for treatment of CAD with EE-BRS in DM patients is limited. We therefore examine the midterm safety and efficacy outcomes of EE-BRS from the ABSORB DM Benelux Study.

## Methods

The ABSORB DM Benelux Study is a prospective international trial in patients with DM treated with the ABSORB family conducted in 18 different EE-BRS experienced centres in The Netherlands, Belgium and Luxembourg. The study was performed in concurrence with the Declaration of Helsinki and approved by the Ethical Committees of all participating centres.

### Study population

The study enrolled DM patients aged ≥ 18 years undergoing PCI with implantation of ≥ 1 EE-BRS for any indication, in a de novo lesion, located in a native non-grafted artery. The exclusion criteria were determined as: pregnancy; patients unable to provide (written) informed consent; known left ejection fraction < 30%; life expectancy < 3 years and inability to take dual antiplatelet therapy (DAPT) for at least 12 months.

### Endpoints and definitions

The endpoints were: major adverse cardiac events (MACE) defined as a composite of all-cause death, any myocardial infarction (MI) and ischemic-driven target vessel revascularization (TVR); target lesion failure (TLF) defined as a composite of cardiac death (CD), target vessel MI and ischemic-driven target lesion revascularization (TLR) and the incidence of definite or probable scaffold thrombosis (ScT).

All-cause death was determined as death to any cause. CD was defined as any death due to immediate cardiac cause like MI, arrhythmia or congestive heart failure. Unwitnessed death, death due to unknown cause, death secondary to cerebrovascular accident or death related to PCI or CABG, were all classified as CD. MI definitions were defined according to the most recent universal definition of MI [23]. TVR was defined as any repeat PCI or CABG of the target vessel of any segment of the target vessel. TLR was defined as any repeat PCI or CABG of the target lesion performed for restenosis or other complication in which the treated segment was located 5 mm proximal and 5 mm distal to the scaffold. A revascularization was considered as ischemic-driven if: (i) angiography showed a diameter stenosis ≥ 50% on quantitative coronary angiography (QCA) and if a single criteria of the following occurred: a positive history of recurrent angina pectoris presumably related to the target vessel or objective signs of ischemia at rest or during exercise test related to the target vessel; (ii) abnormal results of any invasive functional diagnostic test; (iii) presence of a ruptured coronary atherosclerotic lesion on intracoronary imaging evaluation in the presence of clinical symptoms related to an acute coronary syndrome (ACS). Further general definitions were defined as described in the ACC/AHA Clinical Data Standards [24]. ScT was defined according to the Academic Research Consortium [25].

### Percutaneous coronary intervention procedure

The implanted devices are the bioresorbable polymer drug-eluting scaffold ABSORB BVS system and the ABSORB GT1 system (Abbott Vascular, Santa Clara, CA, USA). These devices are composed of poly-l-lactic acid (PLLA) and a polymer coating of poly-dl-lactic-acid (PDLLA), which elutes the active substance everolimus, both of which are completely bioresorbable by the body in a natural 3-year metabolic process [26]. The choice to implant an EE-BRS was at the discretion of the operator. The vessel size, similar to other trials, ranged from 2.50 till 3.75 mm. Predilatation and postdilatation were strongly recommended. Intracoronary imaging by the means of optical coherence tomography (OCT) or intravascular ultrasound (IVUS) were encouraged but not mandatory. Treatment of bifurcations was not endorsed, however in this case provisional T-stenting technique was advised. Additional implantation with metallic DES was accepted at the destined target lesion as bailout when multiple devices were needed and the appropriate EE-BRS size was unavailable. Angiographic success was defined as a < 30% residual stenosis of the target lesion. All patients were prescribed with DAPT for at least 12 months.

### Follow-up and assessment of adverse events

The last follow-up for all patients was performed in February 2018. The average follow-up period was 1.7 years, ranging from 1.0 to 2.8 years. Clinical follow-up was obtained by clinical visits and telephone contact. All reported cardiac adverse events underwent assessment by an independent clinical event committee (CEC) (Diagram BV, Zwolle, The Netherlands). Angiographic evaluation of baseline as well as repeat angiograms in patients with events were analyzed from an independent core lab (Diagram BV, Zwolle, The Netherlands).

### Statistical analysis

The baseline clinical and angiographic characteristics are presented using descriptive statistics. Categorical variables are summarized as frequency and percentages. Continuous variables are summarized as mean and standard deviation. The safety and efficacy outcomes are presented as Kaplan–Meier estimates at 2-years as well as event rates per 100 patient years (PY) with Poisson distribution both given with 95% confidence interval (CI) to adjust for the variable time to follow-up. In addition, a multivariate Cox regression model with adjustment for age, gender, indication for angiography (ACS vs. non-ACS) and insulin-treated DM were performed for both MACE and TLF and were reported in hazard ratio (HR) with 95% CI. Other regression models were performed for relevant factors as proximal vs. distal segment treatment (left main and proximal coronary location—segment number 1, 5, 6, 11 vs. not), pre- and postdilatation, use of intracoronary imaging, total device length and multiple vessel or lesion treatment (≥ 2 vessels/lesions). A *p* value of < 0.05 is considered as statistically significant. All statistical analyses were performed using SPSS version 25 (IBM Corp., Armonk, NY, USA).

## Results

Between April 2015 till March 2017, a total of 150 DM patients and 188 lesions underwent PCI with implantation of EE-BRS. Device implantation success was 100%. Procedural success occurred in all but a single patient where a non-EE-BRS related event occurred; wire distal dissection. Baseline clinical and angiographic characteristics are shown in Tables 1 and 2 respectively.

**Table 1:** Clinical characteristics of the patients at baseline

Baseline clinical characteristic	Patient nr. 150
Age (years)—mean ± SD	64.3 ± 10.4
Sex (male)—no. (%)	108 (72.0)
Race (Caucasian)—no. (%)	140 (93.3)
Body-mass index (kg/m^2^)—mean ± SD; *no.*	29.5 ± 5.1; *148*^a^
Risk factors—no. (%)	
Diabetes mellitus type 1	10 (6.7)
Diabetes mellitus type 2	140 (93.3)
Insulin-dependent diabetes mellitus	47 (31.3)
Diabetes mellitus treated with oral antidiabetic	117 (78.0)
HbA1c (mmol/mol)—mean ± SD; *no.*	55.5 ± 11.5; *42*^a^
Arterial hypertension	104 (69.3)
Hypercholesterolemia	100 (66.7)
Family history of cardiovascular disease	59 (39.3)
Current smoker	35 (23.3)
Medical history—no. (%)	
Previous ACS	41 (27.3)
Previous PCI	37 (24.7)
Previous CABG	8 (5.3)
Previous CVA or TIA	10 (8.7)
Severe chronic renal failure^b^	4 (2.7)
Chronic pulmonary obstructive disease^c^	11 (7.3)
Clinical presentation—no. (%)	
Acute coronary syndrome	73 (48.7)
ST-segment elevation myocardial infarction	18 (12.0)
Non-ST-segment elevation myocardial infarction	29 (19.3)
Unstable angina pectoris	26 (17.3)
Non-acute coronary syndrome	77 (51.3)
Stable angina pectoris	59 (39.3)
Silent ischemia	8 (5.3)
Other	10 (6.7)

Plus–minus values are means ± standard and the curved numbers ^a^ represent the known total of which the variable was calculated. ^b^ Renal insufficiency was defined as estimated glomerular filtration rate of less than 30 ml per minute per 1.73 m^2^ of body surface area (GFR < 30 ml/min/1.73 m^2^). ^c^ Chronic pulmonary obstructive disease was defined as ≥ Gold class II

*ACS* acute coronary syndrome, *PCI* percutaneous coronary intervention, *CABG* coronary artery bypass grafting, *CVA* cerebrovascular accident, *TIA* transient ischemic attack

**Table 2:** Angiographic characteristics of the patients at baseline

Baseline angiographic characteristic	
Patient level analysis	
Number of patients—no	150
Number of treated target lesions—mean ± SD	1.3 ± 0.5
Treated target lesions ≥ 2—no. (%)	30 (20.0)
Number of treated target vessels—mean ± SD	1.1 ± 0.3
Treated target vessels ≥ 2—no. (%)	12 (8.0)
Devices implanted in proximal coronary segment—no. (%)^b^	57 (38.0)
Lesion level analysis	
Number of lesions—no.	188
Coronary artery lesion distribution—no. (%)	
Right coronary artery	57 (30.3)
Left anterior descending artery	89 (47.3)
Circumflex artery	40 (21.3)
Arterial or venous graft	2 (1.1)
Coronary artery lesion characteristics	
Visual estimated diameter stenosis—mean ± SD; no.^c^	85.5 ± 11.9; *181*^a^
Bifurcation—no. (%)	27 (14.4)
Device level analysis	
Number of devices—no.	214
Devices distribution—no. (%)	
ABSORB BVS	130 (60.7)
ABSORB GT1	73 (34.1)
Metallic DES	11 (5.1)
Number of devices per lesion—no. (%)	
1	168 (89.4)
2	16 (8.5)
3	2 (1.1)
4	2 (1.1)
Number of devices per lesion—mean ± SD	1.1 ± 0.5
Diameter device—mean ± SD^d^	3.0 ± 0.4
Inflation pressure—mean ± SD; no.^e^	14.3 ± 2.6; *211*^a^
Total treated length—mean ± SD^d^	29.7 ± 19.0
Procedure level analysis	
Results—no. (%)	
Visual diameter stenosis post-procedural < 30%	185* (100)
Post-procedural TIMI grade 3	186* (100)
Angiographic success	188 (100)
Device implantation success	188 (100)
Procedural success	187 (99.5)
Peri-implantation procedures	
FFR measurement—no. (%)	26 (13.8)
Pre-implantation OCT or IVUS—no. (%)	14 (7.4)
Predilatation—no. (%)	177 (94.1)
Predilatation balloon size—mean ± SD; no.^d^	2.8 ± 0.8; *176*^a^
Predilatation pressure—mean ± SD; no.^e^	14.8 ± 4.0; *174*^a^
Postdilatation—no. (%)	142 (75.5)
Postdilatation balloon size—mean ± SD^d^	3.2 ± 0.5
Postdilatation pressure—mean ± SD^e^	17.3 ± 4.3
Postdilatation balloon size > 0.5 mm than scaffold size—no. (%)	0
Post-implantation OCT or IVUS—no. (%)	15 (8.0)

Shown are the angiographic characteristics of the target lesions of the patients. Plus–minus values are mean ± standard deviation and the curved numbers ^a^ represent the known total of which the variable was calculated. ^b^ Proximal devices were defined as implantation at lesion segments 1,5,6,11. ^c^ Visual estimated diameter stenosis was defined in percent. ^d^ Length of lesions, devices and balloons were measured in millimetre (mm) as was the diameter of the devices. ^e^ Dilatation and inflation pressures were measured in atmosphere (atm)

*DES* drug-eluting stent, *PCI* percutaneous coronary intervention, *TIMI* Thrombolysis in Myocardial Infarction with grade 3 referenced as completely restored flow, *FFR* fractional flow reserve, *OCT* optical coherence tomography, *IVUS* intravascular ultrasound

**Fig. 1:**
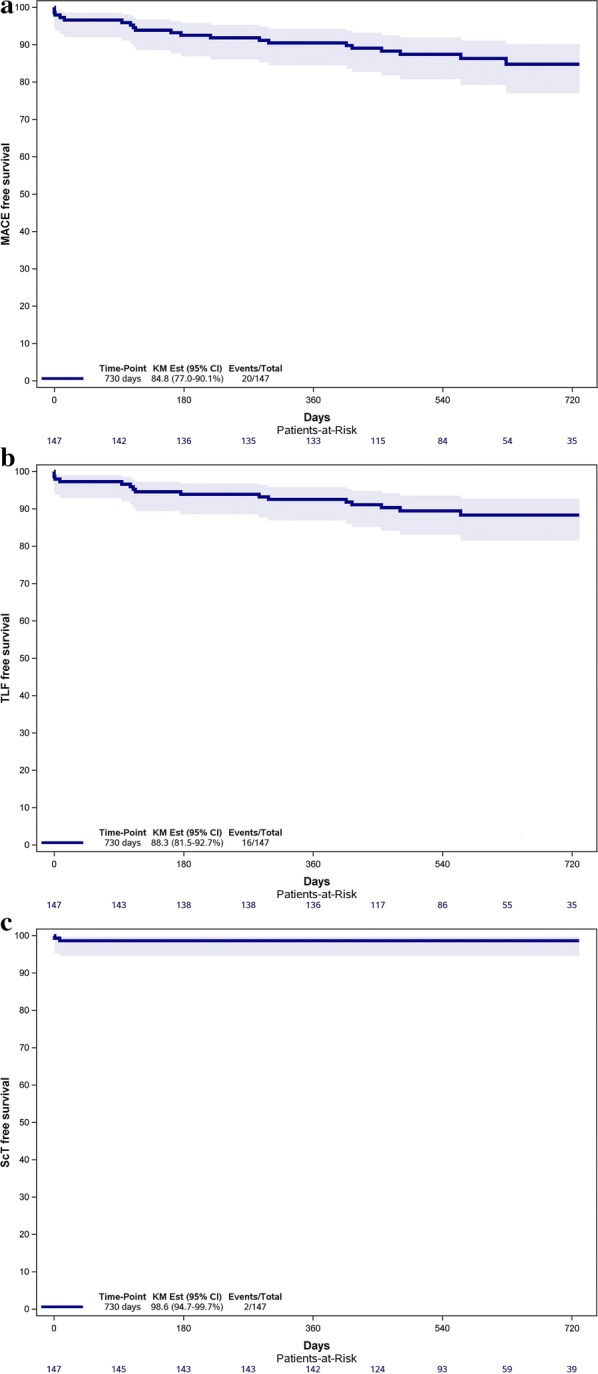
Kaplan–Meier estimates with 95% confidence intervals of **a** major adverse cardiac events, **b** target lesion failure, **c** definite or probable scaffold thrombosis at 2-year follow-up. *MACE* major adverse cardiac events, *TLF* target lesion failure, *ScT* scaffold thrombosis, *KM est* Kaplan–Meier estimate, *CI* confidence interval

**Fig. 2:**
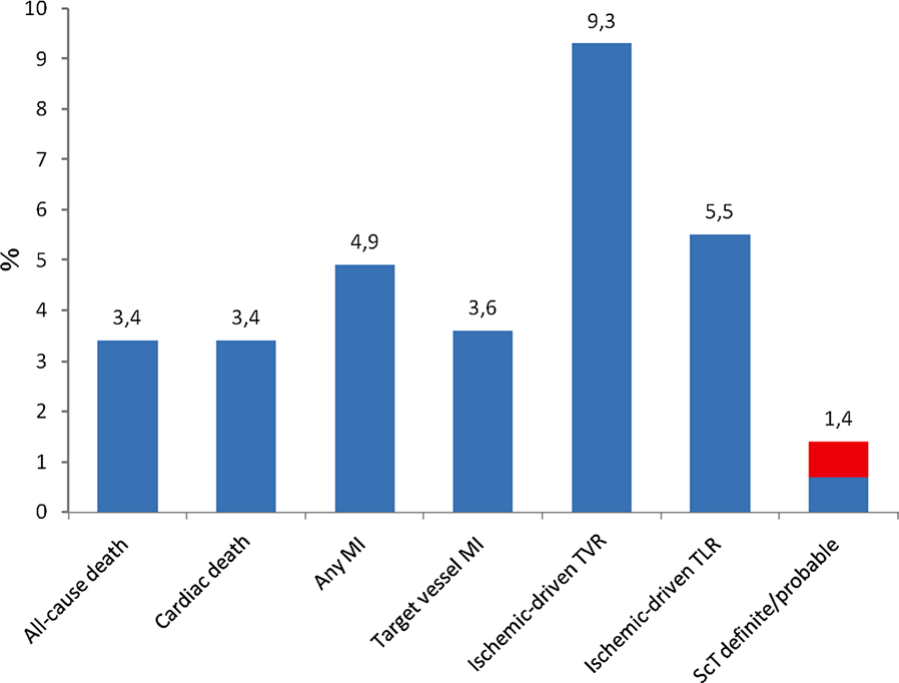
Shown are the 2-year Kaplan–Meier estimates of each of the composites of the endpoints and scaffold thrombosis. The safety outcomes regarding any myocardial infarction, target vessel myocardial infarction and scaffold thrombosis were favourable. For scaffold thrombosis an explicit difference has been made between definite (blue) and probable (red) both accounting for 0.7% of the total 1.4%. There were no occurrences of late or very late definite or probable scaffold thrombosis. *MI* myocardial infarction, *TVR* target vessel revascularization, *TLR* target lesion revascularization, *ScT* scaffold thrombosis

Three patients (2.0%) were lost to follow-up. The clinical outcomes are presented in Table 3. MACE occurred in 15.2% based on Kaplan–Meier estimates or 8.8 events per 100 PY, of which all-cause death occurred in 3.4% (2.1 events per 100 PY), any MI in 4.9% (3.0 events per 100 PY) and ischemic-driven TVR in 9.3% (4.8 events per 100 PY). TLF occurred in 11.7% (7.0 events per 100 PY) of the patients composed out of CD in 3.4% (2.1 events per 100 PY), target vessel MI in 3.6% (2.1 events per 100 PY) and ischemic-driven TLR in 5.5% (3.0 events per 100 PY). Definite or probable ScT was observed in 1.4% (0.8 events per 100 PY) and these 2 events were both classified as early subacute ScT. No events of late or very late ScT were reported. Figure 1 shows the Kaplan–Meier curves of MACE, TLF and ScT while Fig. 2 shows these estimates of the composites of the endpoints for the 2-year follow-up. In a multivariate Cox regression model adjusted with age, gender and indication angiography, DM treated with insulin showed a trend as predictor for both MACE and TLF (HR 2.01; 95% CI 0.80–5.04; p = 0.14 and HR 2.54; 95% CI 0.93–6.97; p = 0.07 respectively), while absence of postdilatation showed a trend as predictor for TLF (HR 0.40; 95% CI 0.15–1.07; p = 0.07) as is presented in Table 4. Other regression models for relevant factors did not prove significant differences.

**Table 3:** Safety and efficacy outcomes at follow-up

Endpoints and clinical events	% (n)	Lower 95% CI	Higher 95% CI	Event rate per 100 PY	Lower 95% CI	Higher 95% CI
MACE^a^	15.2 (20)	0.77	0.90	8.8	5.38	13.61
All-cause death	3.4 (5)	0.92	0.99	2.1	0.67	4.82
Any MI	4.9 (7)	0.90	0.98	3.0	1.21	6.21
Ischemic-driven TVR	9.3 (11)	0.83	0.95	4.8	2.37	8.49
TLF^b^	11.7 (16)	0.82	0.93	7.0	3.97	11.29
CD	3.4 (5)	0.92	0.99	2.1	0.67	4.82
Target vessel MI	3.6 (5)	0.92	0.99	2.1	0.69	4.97
Ischemic-driven TLR	5.5 (7)	0.89	0.97	3.0	1.19	6.12
ScT	1.4 (2)	0.95	1.00	0.8	0.10	3.01
Early: 0–30 days	1.4 (2)					
Acute: ≤ 24 h	0					
Subacute: > 24 h–30 days	1.4 (2)					
Late: 31 days: ≤ 1-year	0					
Very late: > 1-year	0					
Definite	0.7 (1)					
Probable	0.7 (1)					

Clinical outcomes represented as endpoints and clinical events at midterm follow-up. Endpoints and clinical events are presented by Kaplan–Meier estimates at 2-years and in event rates per 100 patient years both given with 95% confidence intervals. Three patients were lost to follow-up

*CI* confidence interval, *PY* patient years, *MACE* major adverse cardiac events, *MI* myocardial infarction, *TVR* target vessel revascularization, *TLF* target lesion failure, *CD* cardiac death, *TLR* target lesion revascularization, *ScT* scaffold thrombosis

^a^Major adverse cardiac event was defined as a composite of all-cause death, any myocardial infarction and ischemic-driven target vessel revascularization

^b^Target lesion failure was defined as a composite of cardiac death, target vessel myocardial infarction and ischemic-driven target lesion revascularization

**Table 4:** Multivariate Cox regression models for major adverse cardiac events and target lesion failure

	Hazard ratio	Lower 95% CI	Higher 95% CI	p-value
A. Variable for outcome MACE				
Gender—female vs. male	1.78	0.58	5.45	0.31
Age at device implantation	1.02	0.98	1.07	0.39
PCI-indication—ACS vs. non-ACS	0.67	0.27	1.62	0.37
Insulin-treated DM—no vs. yes	2.01	0.80	5.04	0.14
B1. Variable for outcome TLF				
Gender—female vs. male	3.44	0.76	15.47	0.11
Age at device implantation	1.03	0.98	1.09	0.27
PCI-indication—ACS vs. non-ACS	0.61	0.22	1.65	0.33
Insulin-treated DM—no vs. yes	2.54	0.93	6.97	0.07
B2. Variable for outcome TLF				
Gender—female vs. male	2.87	0.65	12.69	0.17
Age at device implantation	1.03	0.97	1.09	0.32
PCI-indication—ACS vs. non-ACS	0.64	0.24	1.74	0.39
Postdilatation performed—no vs. yes	0.40	0.15	1.07	0.07

Section A. Multivariate Cox regression model for major adverse cardiac events adjusted with age, gender, PCI-indication and diabetes mellitus treated with insulin at hospital admission. Section B. The same model calculated for target lesion failure (B1) and a model with incorporation of postdilatation (B2). Risk factors are given in hazard ratio’s with 95% confidence intervals with corresponding p-values. A p-value < 0.05 was uphold as formally statistical significant. Insulin-treated diabetes mellitus was identified as variable that showed a trend towards a predictor for major adverse cardiac events. Insulin-treated diabetes mellitus and absence of postdilatation were identified as variables that showed a trend towards a predictor for target lesion failure

*MACE* major adverse cardiac events, *CI* confidence interval, *PCI* percutaneous coronary intervention, *ACS* acute coronary syndrome, *DM* diabetes mellitus, *TLF* target lesion failure

## Discussion

The Benelux ABSORB DM Study, the largest dedicated prospective study examining the safety and efficacy of EE-BRS for percutaneous treatment of CAD exclusively in DM patients for any indication, shows favourable results in this clinically high-risk patient population at midterm follow-up. Indeed, regarding the safety outcomes we observed a low rate of any MI and target vessel MI at 2 years. Importantly, we observed no occurrence of definite or probable ScT events beyond 30 days.

In absence of dedicated trials giving insight in EE-BRS treatment for DM patients at midterm follow-up, comparing our results to other non-exclusive DM EE-BRS studies remains challenging. Several studies have shown favourable outcomes with EE-BRS at short- and long-term follow-up in daily practice [27, 28]. In particular another retrospective study showed favourable outcomes with EE-BRS at 2 years, comparable to modern DES in DM patients [29]. Similarly, other studies, have shown favourable results with EE-BRS at longer follow-up, however these studies were not exclusively focussed on DM patients [30, 31]. However, our data reveals similar clinical outcomes as the everolimus-eluting stent (EES) arm from a pooled database from the SPIRIT and COMPARE trials in DM patients [11]. Indeed, the ischemic-driven TVR was 9.3% in our study vs. 8.3% in the EES arm from the pooled database, while ischemic-driven TLR was identical (5.5% vs. 5.5%). Similarly, the incidence rate of CD (3.4% vs. 2.1%) and any MI (4.9% vs. 4.2%) were quite similar, while the incidence of device thrombosis was also comparable (1.4% vs. 1.1%). This restates that the device-oriented endpoints for both groups might show resemblances in a DM population. Moreover, the patient groups in both trials had similar baseline and angiographic characteristics that improves the degree of comparability. Furthermore, insulin-treated DM was identified as a risk factor for progressing into MACE in the combined SPIRIT and COMPARE trials as was found in this register and an earlier sub-analysis of the ABSORB trials enforcing the believe that insulin-treated DM is a risk factor for EE-BRS treatment as well as for metallic DES [11, 32].

The search for new devices, including resorbable scaffolds, in the treatment of CAD in DM patients originates from previous evidence that consistently reported that DM patients suffer worse outcomes with PCI in comparison to non-DM patients with higher rates of restenosis and stent thrombosis. Furthermore, worse outcomes are also observed when compared to surgical revascularization. The BARI trial indicated that patients who underwent CABG had increased rates of 5 and 10-year survival and decreased rates of MI [12, 13]. The FREEDOM trial enforced that CABG is a superior revascularization strategy in comparison to PCI for DM patients with multivessel disease [14]. However, even CABG, particularly the venous grafts, have limited patency that hardly extents a decade [33], therefore this type of revascularization should be reserved for more advanced aged patients with multivessel disease. Furthermore, within this population of DM patients, newer-generation DES have been associated with better safety and efficacy outcomes as compared to bare metal stents or first-generation DES [34–37]. In addition, a large analysis from a pooled database has shown that in the DES era, clinical outcomes after PCI in DM patients are highly dependent on lesion complexity at baseline [38]. Simple lesions are associated with similar efficacy outcomes as compared to non-DM patients, while DM patients with complex lesions have significantly higher rates of repeat revascularization after DES implantation than non-DM patients. This data suggests that PCI may have favourable results in a well selected group of patients with DM, provided the extent of disease is less complex and thus is consistent with the results from the SYNTAX trial [39]. Henceforth, the principle of bioresorbable scaffolds becomes captivating as it opens the possibility for repetitive revascularization prolonging the time interval where CAD can still be treated with PCI especially in DM patients who in general present with CAD at younger age. In this perspective, the results observed in our study are promising.

Nonetheless, the issue concerning the safety outcomes of the EE-BRS remains controversial given the 2-year AIDA trial results and 3-year outcomes of the ABSORB II and III that in particular proved disadvantageous safety results for EE-BRS in comparison to EES [40–43]. The main reasons for these compromised safety outcomes were recognized as undersizing, under expanding or geographic mismatching of the scaffold at target lesion, increased strut thickness of the EE-BRS with loss of vessel diameter along with distal device implantation, asymmetric or heterogeneous degradation, neoartherosclerosis, restenosis and hemodynamically altered blood flow variation with higher thrombogenicity augmented in the absence of DAPT [44–47]. To counteract this ominous complications, the pre-dilatation, sizing and post-dilatation (PSP) technique has been introduced as a recommendation for performing aggressive predilatation to improve vessel compliance, enabling full scaffold expansion; appropriate vessel sizing, avoidance of very small vessels in which EE-BRS has excessive surface area coverage and polymer volume occupancy; and routine, aggressive postdilatation with slightly oversized noncompliant balloons at high pressure to ensure maximize scaffold dimensions, reduce shear stress and avoid acute malapposition [48].

The reasons for the low incidence of target vessel MI after the first year (and thus also for absence of very late ScT) in this register may be explained by the increased experience of the operators implanting the EE-BRS even in absence of a well established PSP-protocol. To a greater extent, absence of postdilatation did also show a trend as predictor for TLF. Indeed, another study with usage of EE-BRS in DM patients showed that a good implementation technique is associated with improved clinical outcomes in this specific population [49]. On the contrary, these low occurrences in our study are not to be explained by the use of intracoronary imaging which was implemented rather infrequently (pre-implantation 7.4% and post-implantation 8.0%). Derived from this sentiment, it is conceivable that even further improvement in clinical endpoints might be obtained if intravascular imaging is more routinely performed. Furthermore, in a meta-analysis of multiple EE-BRS trials in a 2-year follow-up, a bimodal incidence rate of ScT was reported with a total ScT mainly being composed of early subacute and very late ScT [50]. The majority of very late ScT occurred in de absence of DAPT emphasizing the potential beneficial role of this medical regiment. At the moment, long DAPT regimens are also recommended for DM patients treated with metallic DES [51, 52]. In this register, the advantageous role of prolonged DAPT could not be reproduced as, in a total of 6 additional events after 1-year, 3 events occurred under DAPT and 3 events without DAPT. The prolonged prescription of P2Y_12_ antagonists was 74.8% at 1 year and 56.5% at 1.7 year taking into account the bleeding risk of each patient individually with DAPT score.

Following from above, we believe that EE-BRS has similar clinical outcomes to current metallic DES that, considering its bioresorbable nature, could offer an alternative to metallic DES for treatment of non-complex CAD in DM patients, especially if performed by experienced operators who are familiar with the PSP-technique for optimizing safety results. Insulin-dependent DM patients of elderly age, with diffuse coronary disease, show potentially lower benefit from the resorbable aspect of the scaffold and considering the higher bleeding risk under prolonged DAPT regiments may be unsuitable candidates for EE-BRS treatment. Furthermore, development of new generation resorbable scaffolds, with thinner strut diameters and with more homogenous resorption patterns, are paramount. If these device orientated factors could be counteracted in the future, EE-BRS might obtain a valuable place in the treatment of CAD in a group of well selected DM patients.

## Limitations

This study has the general intrinsic limitations of a single-arm prospective register with no intrinsic comparison group. The study population was numerically smaller than foreseen due to the effectuated stop in clinical utilization of the EE-BRS. Furthermore, PSP-technique for scaffold implantation was not part of our implantation protocol however all the selected centres as well as the operators had extensive experience with the EE-BRS device. Finally, the patient follow-up period ranged from 1.0 to 2.8 years, which may lead to an inaccurate estimation of the clinical outcomes at 2-year analysis, therefore to give a better evaluation we also presented the results in event rates per 100 PY. Considering the expected 3-year resorption time of the EE-BRS, our study does not provide insights into the outcomes of this resorbable scaffold beyond its resorption time, however we chose to present our results now in order to share our data with the interventional community as the debate over the future and the need for resorbable scaffolds is ongoing.

## Conclusion

The results of this dedicated multicentre prospective study show that treatment of non-complex CAD with EE-BRS in DM patients show favourable safety and efficacy outcomes, comparable to those of modern metallic DES when historically compared. However this treatment should be considered and performed by experienced and adequately trained operators familiar with certain device implantation requirements. Within the limitations of our study, EE-BRS and possibly newer and more sophisticated generation of scaffolds with thinner strut diameters may open new horizons in treatment of CAD in DM patients.

## Abbreviations

ACS: acute coronary syndrome
CABG: coronary artery bypass grafting
CAD: coronary artery disease
CD: cardiac death
CEC: clinical event committee
CI: confidence interval
DAPT: dual antiplatelet therapy
DES: drug-eluting stent
DM: diabetes mellitus
EE-BRS: everolimus-eluting bioresorbable scaffolds
EES: everolimus-eluting stent
HR: hazard ratio
IVUS: intravascular ultrasound
MACE: major adverse cardiac events
MI: myocardial infarction
OCT: optical coherence tomography
PCI: percutaneous coronary intervention
PDLAA: poly-dl-lactic-acid
PLLA: poly-l-lactic acid
PSP: pre-dilatation, sizing and post-dilatation
PY: patient years
QCA: quantitative coronary angiography
ScT: scaffold thrombosis
TLF: target lesion failure
TLR: target lesion revascularization
TVR: target vessel revascularization
